# CHANGES IN SOCIAL SUPPORT AND THE INFLUENCE OF DRIVING CESSATION

**DOI:** 10.1093/geroni/igz038.2767

**Published:** 2019-11-08

**Authors:** Brittany M King

**Affiliations:** Florida State University, Tallahassee, Florida, United States

## Abstract

Driving cessation has been shown to be a potentially isolating transition in life, with important implications for mental health, social isolation, and social support. Older adults who live alone are vulnerable to social isolation in the context of driving cessation. Although some research has examined the association between driving cessation and certain kinds of social engagement activities, no research has specifically examined changes in social support, particularly among older adults most vulnerable to social isolation – those who live alone . The present study addresses this gap, using data drawn from the 2006-2014 waves of the Health and Retirement Study (HRS) to examine how social support changes in the context of driving cessation among older adults who live alone (N=412). This study specifically focuses on instrumental and emotional social support, and how different sources of the support (children, friends, and other family) are influenced by loss of driving. I use a series of ordinary least squares regression (OLS) to examine four-year changes in various forms of social support among those who live alone, comparing those who lose the ability to drive relative to their continuously driving counterparts. Preliminary results indicate that driving cessation is associated with decline in perceived instrumental support of friends (-0.984, p<01) for older adults who live alone. However, these effects did not extend to children or other family members. These results suggest that loss of driving may perpetuate vulnerabilities facing individuals who live alone by leading to lower levels of perceived support from non-family members.

